# Is Abnormal Urine Protein/Osmolality Ratio Associated with Abnormal Renal Function in Patients Receiving Tenofovir Disoproxil Fumarate?

**DOI:** 10.1371/journal.pone.0149562

**Published:** 2016-02-12

**Authors:** Jasmine R. Marcelin, Melody L. Berg, Eugene M. Tan, Hatem Amer, Nathan W. Cummins, Stacey A. Rizza

**Affiliations:** 1 Department of Medicine, Division of Infectious Diseases, Mayo Clinic, Rochester, MN, United States of America; 2 Department of Medicine, Division of Infectious Diseases, Mayo Clinic Rochester, MN, United States of America; 3 Department of Internal Medicine, Mayo Clinic, Rochester, MN, United States of America; 4 Department of Medicine, Division of Nephrology & Hypertension, Mayo Clinic, Rochester MN, United States of America; University of São Paulo School of Medicine, BRAZIL

## Abstract

**Background:**

Risk factors for and optimal surveillance of renal dysfunction in patients on tenofovir disoproxil fumarate (TDF) remain unclear. We investigated whether a urine protein-osmolality (P/O) ratio would be associated with renal dysfunction in HIV-infected persons on TDF.

**Methods:**

This retrospective, single-center study investigated the relationship between parameters of renal function (estimated glomerular filtration rate (eGFR) and P/O-ratio) and risk factors for development of kidney dysfunction. Subjects were HIV-infected adults receiving TDF with at least one urinalysis and serum creatinine performed between 2010 and 2013. Regression analyses were used to analyze risk factors associated with abnormal P/O-ratio and abnormal eGFR during TDF therapy.

**Results:**

Patients were predominately male (81%); (65%) were Caucasian. Mean age was 45.1(±11.8) years; median [IQR] TDF duration was 3.3 years. [1.5–7.6]. Median CD4+ T cell count and HIV viral load were 451 cells/μL [267.5–721.5] and 62 copies/mL [0–40,150], respectively. Abnormal P/O-ratio was not associated with low eGFR. 68% of subjects had an abnormal P/O-ratio and 9% had low eGFR. Duration of TDF use, age, diabetes and hypertension were associated with renal dysfunction in this study. After adjustment for age, subjects on TDF > 5 years had almost a four-fold increased likelihood of having an abnormal P/O-ratio than subjects on TDF for < 1yr (OR 3.9; 95% CI 1.2–14.0; p = 0.024).

**Conclusion:**

Abnormal P/O-ratio is common in HIV-infected patients on TDF but was not significantly associated with low eGFR, suggesting that abnormal P/O-ratio may be a very early biomarker of decreased renal function in HIV infected patients.

## Introduction

Chronic kidney disease in persons infected with HIV is an important part of the changing epidemiology of the disease. Prior to the introduction of combined antiretroviral therapy (cART), HIV-associated nephropathy (HIVAN) was the most common cause of kidney disease in HIV. [1] However, certain components of cART can also cause tubular, glomerular or interstitial damage and nephrolithiasis, associated with acute and chronic kidney disease. [2, 3] Tenofovir disoproxil fumarate (TDF) is one of the most commonly prescribed antiretrovirals in the United States. [4] TDF, an oral prodrug of tenofovir (TFV), is a nucleotide analog of adenosine monophosphate with activity against HIV-1 nucleoside reverse transcriptase. TDF is well-tolerated, efficacious, easy to administer, and is a recommended component of the majority of first-line cART regimens in the Department of Human Health and Services (DHHS) guidelines for the management of patients with HIV. [5]

However, TDF can be associated with renal dysfunction. Reports of tubular damage, Fanconi syndrome, interstitial nephritis or overall decrease in estimated glomerular filtration rate (eGFR) have been associated with its use. [3, 6–8] The proposed mechanism of renal injury is an independent direct cytotoxic effect by proximal tubular accumulation of the drug and prevention of organic acid transport.[6, 9] After ingestion, TDF undergoes rapid plasma metabolism to TFV, which is subsequently phosphorylated to its active form TFV-diphosphate within cells. Therefore, it is the circulating plasma TFV which then undergoes both glomerular filtration and tubular secretion in the kidneys by the organic acid transporters (OAT). [10]

Rats exposed to high doses of TDF have been shown to develop renal cortical damage with glomerular and proximal tubule distortion. [11] TDF may also lead to mitochondrial damage in proximal tubules, with treated rats developing giant mitochondria, cristae disruption and amorphous deposits within the mitochondrial matrix. [11, 12] The OAT type 1 transporter has been specifically implicated in TDF/TFV transport into tubules, with OAT1 knockout mice being protected from TDF/TFV nephrotoxicity. [13] Additionally, major renal mitochondrial antioxidant enzyme function is significantly impacted, as evidenced by decreased activity of succinate dehydrogenase, glutathione reductase, superoxide dismutase, and glutathione peroxidase. [11]

Certain factors may exacerbate the nephrotoxic effect of TDF. In rat models, vitamin D deficiency in TDF-treated subjects has been demonstrated to worsen proteinuria, increase oxidative stress, and contribute to metabolic syndrome and hypertension (through upregulation of the renin-angiotensin-aldosterone system). [14] Furthermore, administration of TDF with HIV protease inhibitors has been demonstrated to increase the serum concentrations of TDF by 30%, thus increasing the potential for nephrotoxicity. [9]

Studies have demonstrated that the incidence of TDF-induced nephrotoxicity (due to increased renal exposure to TFV) increases in patients with advanced age, low body weight, increased baseline serum creatinine, advanced HIV (defined as CD4+ T cell count <200 cells/μL), and other comorbidities such as hypertension, diabetes and cardiovascular disease. [4, 12, 15–18] However, this nephrotoxicity can be at least partially reversible, with urinary protein levels decreasing rapidly and slower decline of eGFR after discontinuation of TDF. [12, 19]

Conversely, some studies have demonstrated that long-term TDF use did not induce nephrotoxicity,[20] and still others have conceded that though there may be clinically significant renal dysfunction with TDF use, the benefits of viral suppression outweigh these potential risks.[15, 21–23] Regardless of the final opinion about TDF-associated nephrotoxicity, most agree that renal function should be monitored closely when on TDF therapy. [3, 23, 24] The Infectious Diseases Society of America (IDSA) recently updated guidelines for management of chronic kidney disease in HIV-infected patients; in this document, experts endorsed at least bi-annual creatinine measurement and quantitative measurement of proteinuria at least annually.[25]

However, other professional societies differ in terms of recommendations. For example, the 2014 DHHS guidelines recommend a urinalysis every 6 months if on TDF.[5] The 2010 World Health Organization guidelines recommend creatinine clearance calculation before initiation and every 6 months while on TDF.[26] Finally, the 2014 guidelines from the International Antiviral Society do not specifically address renal function monitoring while on TDF.[27] Therefore, it remains unclear which screening modality is most appropriate for detecting changes in renal function on TDF.

Proteinuria is one marker of renal disease and can be assessed in different ways. The urine protein-creatinine ratio is used by most institutions to approximate the 24-hour protein excretion; however, limitations include fluctuations in urine creatinine concentrations at various times of day as well as different patterns of excretion of urine protein and creatinine (and therefore variable protein-creatinine ratios) depending on the type of kidney injury. [28] The P/O-ratio was first described for assessing abnormal proteinuria in quantitative urinalysis in 1993 by Wilson and Anderson. [28] They found that the P/O-ratio was superior to protein-creatinine ratio in predicting abnormal 24 hour proteinuria, with 96% sensitivity and 93% specificity. [28] As a result, our institution moved to using P/O ratio for routine urinalysis, using other measures of proteinuria such as protein-creatinine ratio, 24hr proteinuria, and urine beta-2 microglobulin for more focused nephrology evaluations.

The objective of our study was to define the prevalence of abnormal urine P/O-ratio and subsequently determine whether the urine P/O-ratio associates with abnormal estimated GFR in patients receiving TDF therapy as a component of cART. Additionally, we aimed to identify risk factors for renal dysfunction in this population.

## Patients and Methods

### Human Subjects and Ethics Statement

Human subjects were involved indirectly through retrospective chart review. All subjects had previously signed releases of information authorizing their charts to be reviewed for medical research purposes before the study was conducted. All records reviewed were kept confidential, and patient-identifiable information was de-identified prior to analysis. The minimal risk, HIPAA-waiver study received approval from the Mayo Clinic Institutional Review Board (IRB#13–003409).

### Study Setting, Design and Subject Selection

This was a retrospective, single-center, cross-sectional chart review study involving patients seen in the HIV clinic at Mayo Clinic, Rochester, MN. The patients served by this clinic include those living in Olmsted County and surrounding counties, as well as many who travel from their hometowns throughout the US and the rest of the world to seek HIV care in Rochester. Medical records of patients within the Mayo Clinic HIV Database from January 1, 2010 to December 31, 2013 were reviewed for eligible subjects.

Subjects were included in the study if they met the following criteria: (1) adults ≥18 years old, (2) diagnosed with HIV-1 infection, (3) were on a TDF-containing cART regimen between January 1, 2010 and December 31, 2013, and (4) had at least one documented urinalysis and two serum creatinine levels within the study period. Subjects were excluded from consideration if they (1) were less than 18 years old, (2) had a previous diagnosis of chronic kidney disease or proteinuria identified prior to the study period, regardless of TDF use (3) had documented receipt of additional potentially nephrotoxic medications during the period of abnormal urinalysis or renal insufficiency, or (4) had no documented urinalysis between January 1, 2010 and December 31, 2013.

### Chart Review

Demographic and clinical characteristics, P/O-ratios, serum creatinine, eGFR, and comorbidities (diabetes, hypertension, hyperlipidemia), were collected using a standardized case report form. All TDF-monitoring P/O-ratios obtained during the study period were included. The first abnormal P/O-ratio encountered was used to determine the prevalence of abnormal P/O-ratio among subjects on TDF between 2010 and 2013. The date of the last urinalysis between 2010 and 2013 was used in the calculation of duration of TDF therapy for all study subjects. Additional information collected included concurrent use of non-nucleotide reverse transcriptase inhibitors (NNRTIs), protease inhibitors (PIs) or integrase inhibitors, hepatitis B and C co-infection status, presence of concomitant nephrotoxic medications (nonsteroidal antiinflammatory drugs [NSAIDs], sulfamethoxazole/trimethoprim, acyclovir, valacyclovir, valganciclovir), presence of concomitant renoprotective medications (ACE inhibitors and angiotensin receptor blockers), CD4+ T-cell count at beginning of study period, and HIV viral load > 100,000 copies/ml at beginning of the study period.

### Definitions and Measurements

Chronic kidney disease is defined as a functional or structural kidney abnormality present for ≥3 months, with diagnostic criteria including eGFR (as calculated by the Modification of Diet in Renal Disease (MDRD) equation) <60 mL/min/1.73 m^2^ on two separate readings at least 3 months apart and/or persistent proteinuria. [29] The P/O-ratio is automatically reported in the electronic medical record as part of every urinalysis obtained at our institution, based on the equation as follows:

$$protein:osmolality\;ratio=\frac{protein\;\left(mg/L\right)}{osmolality\;\left(mOsm/kg\right)}$$

The normal P/O-ratio was defined as ≤0.12, which corresponds to a normal protein-creatinine ratio of ≤0.05 [28]. Predicted 24-hr proteinuria is an automatic value provided in the routine urinalysis. It is a calculated estimate derived from the P/O ratio with the following equation[28]:

$$Females:\;mg/24hr\;protein\;=10^{0.908[{log}_{10}protein/osmolality]+[2.8254\pm 0.6073]}$$

$$Males:\;mg/24hr\;protein\;=\;10^{0.953[{log}_{10}protein/osmolality]+[2.9739\pm 0.498}$$

In this study, we classified predicted 24-hr proteinuria as normal/mild (<150mg/24hrs), moderate (150-500mg/24hrs) or severe (>500mg/24hrs), according to the Kidney Disease: Improving Global Outcomes (KDIGO) guidelines[30]. Abnormal eGFR was defined as <60ml/min/1.73m^2^. The eGFR was calculated using the National Kidney Foundation endorsed four variable MDRD equation as follows: [31]

eGFR (mlmin173m2)=175×((Scr)-1.154)×(Age)-0.203×(0.742 if female)×(1.212 if African American)

P/O-ratios are evaluated as part of urinalyses collected on (at least) an annual basis for subjects on TDF-containing regimens, according to 2009 DHHS guidelines (which were the most current at the start of the time period being investigated). [5] Any other urinalyses collected at other times during the period 2010–2013 (e.g. in patients who developed dysuria or other urinary symptoms) were not included as these were not done specifically for designated TDF monitoring according to the protocol. Similarly, the only creatinine values considered were those associated with the designated TDF-monitoring urinalyses. Major outcomes of interest include prevalence of abnormal P/O-ratio associated with abnormal eGFR.

### Statistical analysis

Statistical analyses were performed using JMP software version 10.0 (SAS Institute Inc., Cary, NC, 1989–2007).Descriptive statistics were used to describe baseline clinical characteristics of the subjects. Factors associated with abnormal P/O ratio, or abnormal eGFR during TDF therapy were determined using a univariate logistic regression analysis with Chi-squared and Fisher’s exact test as appropriate to calculate odds ratios with 95% confidence intervals. Factors with significant odds of association with abnormal P/O ratio or abnormal eGFR were included in a multivariable logistic regression analysis. P values were calculated with likelihood ratios and values <0.05 were considered statistically significant. Sensitivity analyses were performed where appropriate to exclude potential confounding factors.

## Results

Characteristics of the 117 subjects included in the study are listed in Table 1. 95 (81%) of the study subjects were male and 76 (65%) were Caucasian. The mean age of subjects was 45.1 ±11.8 years and median (IQR) TDF duration was 3.3 years (1.5, 7.6). Median (IQR) CD4+ T-cell count and HIV viral load at study initiation were 451 (268, 722) cells/μL and 62 (0, 40150) copies/mL, respectively. 80 (68%) subjects had an abnormal P/O-ratio with a median (IQR) P/O-ratio of 0.14 (0.09, 0.25) and 11 (9%) had abnormal eGFR between 2010 and 2013. Using the calculated predicted 24hr proteinuria categories, 53(45.3%), 46(39.3%) and 18(15.4%) subjects had mild, moderate and severe proteinuria, respectively at some point while on TDF between 2010 and 2013. When testing the distribution of predicted 24hr proteinuria values for a difference between those with and without an abnormal eGFR, there was a borderline association of higher predicted 24hr proteinuria values with abnormal eGFR, though this result was not statistically significant (p = 0.07). However, using combined predicted 24hr proteinuria categories, moderate/severe proteinuria was significantly associated with reduced eGFR (OR 10.7 11.3–86.6, p = 0.01) compared to mild proteinuria. Further subgroup analysis did not reliably differentiate outcomes between moderate and severe categories. This may represent in part the small size of the severe category.

**Table 1:** Baseline characteristics of study subjects.

Study subject characteristics	Number (%) or Median [IQR] or Mean ±SD [N = 117]
Sex—Male	95 (81%)
Age	45.1 ±11.8
Race: African American	22 (19%)
Race: American Indian	1 (0.01%)
Race: Asian	5 (0.04%)
Race: Caucasian	76 (65%)
Race: Other*	13 (11%)
TDF duration (years) at last urinalysis	3.3 [1.5–7.6]
Median P/O-ratio	0.14 [0.09–0.25]
Any abnormal P/O-ratio in study period	80 (68%)
Median predicted 24-hr proteinuria (mg/24hrs)	137 (91.5, 249.5)
Any eGFR <60 mL/min/1.73m^2^	11 (9%)
Concurrent Protease Inhibitor use	53 (45%)
Concurrent NNRTI use	77 (66%)
Concurrent INSTI use	11 (9%)
Median CD4+ T cell count(cells/μL)	451 [267.5–721.5]
Any with CD4+ T cell count 200 cells/μL	22 (18.8%)
Median HIV viral load	62 [0–40150]
Any with HIV viral load >100,000 copies/mL	24 (20.5%)
Diabetes Mellitus	11 (9%)
Hypertension	28 (24%)
Hyperlipidemia	39 (33%)
HCV co-infection	16 (14%)
HBV co-infection	14 (12%)
Concurrent TMP/SMX use	24 (21%)
Concurrent NSAID use	17 (15%)
Concurrent Antiviral use^a^	10 (9%)

*Other—patient listed other in demographic portion of medical record

^a^Acyclovir, Valacyclovir, Valganciclovir, Ganciclovir.

Abbreviations: IQR, inter-quartile range; SD, standard deviation; TDF, tenofovir disoproxil fumarate; P:O, protein-osmolality ratio; eGFR, estimated glomerular filtration rate; NNRTI, non-nucleoside reverse transcriptase inhibitor; INSTI, integrase inhibitor; VL, HIV viral load; HCV, hepatitis C virus; HBV, hepatitis B virus; TMP/SMX, Trimethoprim/Sulfamethoxazole; NSAID, nonsteroidal anti-inflammatory drug.

Tables 2 and 3 describe the risk factors of the patients taking TDF and the relationships of these risk factors to a P/O-ratio ≥0.12 and eGFR<60ml/min/1.73m^2^ respectively. After adjusting for age, subjects on TDF >5 years had almost a four-fold increased association with an abnormal P/O-ratio than people on TDF for <1yr (OR 3.9; 95% CI 1.2–14.0; p = 0.024). When a sensitivity analysis was performed excluding diabetic subjects and treating duration of tenofovir use as a continuous variable, there was a difference in association with abnormal P/O-ratio per 1 year increment on tenofovir (OR 1.2 95%CI 1.02–1.41, p = 0.019 vs OR 1.15 95%CI 0.98–1.37, p = 0.080). A 10-year increase in age above the mean was associated with having an abnormal P/O-ratio (OR 1.42, 95% CI 1.0–2.1, p = 0.048) and abnormal GFR (OR 2.1, 95% CI 1.3–3.8; p = 0.048).

**Table 2:** Risk Factors of patients and relationship to any abnormal P/O-ratio within study period.

Risk Factor	Abnormal (%) or Mean ±SD [N = 80]	Normal (%) or Mean ±SD [N = 37]	OR	95% CI	p*
Sex—Male	64 (80)	31 (84)	0.77	0.28, 2.17	0.63
Age	46.5±12	41.9±10.9	1.42	1.00, 2.06	0.05
Race—African American	16 (20)	6 (16)	0.77	0.28, 2.17	0.63
eGFR<60ml/min/1.73m^2^	10 (13)	1 (2.7)	5.14	0.63, 41.77	0.17
TDF duration <1yr^a^	11 (14)	11 (30)	REF	REF	REF
TDF duration 1-5yrs^a^	35 (44)	19 (51)	1.77	0.64, 4.92	0.27
TDF duration >5yrs^a^	34 (43)	7 (19)	3.97	1.20, 14.01	0.02
Concurrent PI use	39 (49)	14 (38)	1.56	0.71, 3.46	0.27
Concurrent NNRTI use	51 (64)	26 (70)	0.74	0.32, 1.72	0.49
Concurrent INSTI use	7 (9)	4 (11)	0.79	0.22, 2.89	0.74
CD4+T cell count <200 cells/μL at study initiation	16 (20)	8 (22)	0.91	0.35, 2.36	0.84
HIV VL >100,000 copies/mL during study	29 (36)	18 (49)	1.67	0.76, 3.67	
Diabetes Mellitus	11 (14)	0 (0)	n/a^b^	n/a^b^	0.02
Hypertension	23 (29)	5 (14)	2.58	0.90, 7.45	0.07
Hyperlipidemia	28 (35)	11 (30)	1.27	0.55, 2.95	0.57
HCV co-infection	13 (16)	3 (8)	2.20	0.59, 8.24	0.39
HBV co-infection	11 (14)	3 (8)	1.81	0.47, 6.91	0.54
Concurrent TMP/SMX use	16 (20)	8 (22)	0.91	0.35, 2.36	0.84
Concurrent NSAID use	12 (15)	5 (14)	1.13	0.37, 3.48	0.83
Concurrent Antiviral use^c^	6 (8)	4 (11)	0.67	0.18, 2.52	0.72

^a^After adjusting for age

^b^ n/a because ALL subjects with diabetes mellitus had abnormal P:O ratio

^c^Acyclovir, Valacyclovir, Valganciclovir, Ganciclovir

*p values were calculated using Likelihood ratio

Abbreviations: SD, standard deviation; OR, odds ratio; CI, confidence interval; TDF, tenofovir disoproxil fumarate; PI, protease inhibitor; NNRTI, non-nucleoside reverse transcriptase inhibitor; INSTI, integrase inhibitor; VL, HIV viral load; HCV, hepatitis C virus; HBV, hepatitis B virus; TMP/SMX, trimethoprim/sulfamethoxazole; NSAID, nonsteroidal anti-inflammatory drug; yrs, years

**Table 3:** Risk Factors of patients and relationship to any abnormal eGFR within study period.

Risk Factor	Abnormal (%) or Mean ±SD [N = 11]	Normal (%) or Mean ±SD [N = 106]	OR	95% CI	p*
Sex—Male	10 (91)	85 (80)	0.41	0.05, 3.34	0.69
Age	54.3±11.4	44.0±11.4	2.09	1.25, 3.82	0.05
Race—African American	10 (91)	85 (80)	0.41	0.05, 3.34	0.69
Abnormal P/O-ratio	10 (91)	70 (66)	5.14	0.63, 41.77	0.17
TDF duration <1yr^a^	1 (8)	21 (20)	REF	REF	REF
TDF duration 1-5yrs^a^	8 (67)	46 (44)	2.35	0.34, 47.1	0.42
TDF duration >5yrs^a^	3 (25)	38 (36)	0.46	0.03, 10.9	0.57
Concurrent PI use	6 (50)	47 (45)	1.23	0.37, 4.08	0.73
Concurrent NNRTI use	7 (58)	70 (67)	0.70	0.21, 2.36	0.56
Concurrent INSTI use	2 (18)	9 (8)	4.04	0.91, 18.0	0.09
CD4+T cell count <200 cells/μL at study initiation	3 (25)	21 (20)	1.33	0.33, 5.36	0.71
HIV VL >100,000 copies/mL during study	3 (27)	44 (42)	1.89	0.48, 7.54	0.36
Diabetes Mellitus	0 (0)	11 (10)	0.00	n/a^b^	0.60
Hypertension	6 (50)	22 (21)	3.77	1.11, 12.9	0.03
Hyperlipidemia	5 (42)	34 (32)	1.49	0.44, 5.04	0.52
HCV co-infection	1 (8)	15 (14)	0.55	0.066, 4.54	1.00
HBV co-infection	2 (17)	12 (11)	1.55	0.30, 7.93	0.64
Concurrent TMP/SMX use	3 (23)	21 (20)	1.33	0.33, 5.36	0.71
Concurrent NSAID use	1 (8)	16 (15)	0.51	0.06, 4.19	1.00
Concurrent Antiviral use^c^	0 (0)	10 (10)	0.00	n/a	1.00

^a^ After adjusting for age

^b^ n/a because ALL subjects with diabetes mellitus had normal eGFR

^c^Acyclovir, Valacyclovir, Valganciclovir, Ganciclovir

*p values were calculated using Likelihood ratio

Abbreviations: eGFR, estimated glomerular filtration rate; SD, standard deviation; OR, odds ratio; CI, confidence interval; TDF, tenofovir disoproxil fumarate; PI, protease inhibitor; NNRTI, non-nucleoside reverse transcriptase inhibitor; INSTI, integrase inhibitor; VL, HIV viral load; HCV, hepatitis C virus; HBV, hepatitis B virus; TMP/SMX, trimethoprim/sulfamethoxazole; NSAID, nonsteroidal anti-inflammatory drug.

All 11 diabetic subjects had an abnormal P/O-ratio. Subjects with hypertension were more likely to have abnormal eGFR (OR 3.8, 95% CI 1.1–12.9; p = 0.026). When a sensitivity analysis was performed, excluding diabetic subjects did not have a significant impact on the association of abnormal eGFR and age or hypertension, nor did it have an impact on the association of abnormal P/O-ratio and duration of TDF >5 years. However, exclusion of diabetics eliminated the significant association between abnormal P/O-ratio and age (OR 1.32 95%CI 0.94–1.91, p = 0.115). The sensitivity analysis also eliminated the trend to significance between abnormal P/O-ratio and hypertension (OR 1.77 95%CI 0.62–5.88, p = 0.292). Abnormal P/O-ratio was not significantly associated with abnormal eGFR in this cross-sectional study (p = 0.17), suggesting that in order to adequately monitor renal function in TDF treated patients, both functional markers (e.g. eGFR) and proteinuria assessments are needed.

## Discussion

Increasing age was significantly associated with abnormal renal function on TDF as determined by P/O-ratio and eGFR, which corresponds with findings from previous studies that TDF-containing regimens should be used with caution in older subjects or those with diabetes.[4, 15]. This may be due to a higher prevalence of pre-existing CKD in these populations or due to higher susceptibility. Very few subjects developed abnormal eGFR between 2010 and 2013; though advanced HIV has been shown to be a risk factor for abnormal renal function, [12] this effect was not seen here given that most of our patients were immunologically and virologically controlled on their TDF regimens. However, the duration of the TDF regimen is interesting given that we were able to show that subjects with abnormal P/O-ratios were more likely to have been on TDF for >5 years. Similarly, a prospective pharmacokinetic study nested in the Women’s Interagency HIV study recently demonstrated a strong association between longer tenofovir exposure and declining renal function, with strongest association between 3.5-7years on TDF. [32] In a study of 24 patients on cART who discontinued TDF therapy due to acute kidney failure, patients who were treated with TDF for shorter periods had faster renal recovery than those treated for longer durations. [33] A more recent study of TDF used as HIV pre-exposure prophylaxis (PrEP) followed patients on TDF-containing PrEP regimens for up to 36 months and found small (but infrequently clinically significant) declines in eGFR. [34]

Although the results of our study do not indicate that an elevated P/O-ratio is associated with a high prevalence of low eGFR in HIV infected patients on TDF, the association of TDF duration and renal abnormalities can also be viewed as a confounder of duration of HIV infection. Patients who have had HIV for more years, especially those with viremia, were more likely to have underlying HIVAN, and in these situations, it would be difficult to identify whether or not renal dysfunction in these patients was secondary to HIVAN or antiretrovirals. It should be noted that our study was cross sectional and follow up was limited. The presence of an abnormal P/O could be an early presentation of chronic renal damage that would result in decreasing GFR with prolonged therapy. More studies are needed to better identify the cause of kidney diseases and their natural history in patients treated with TDF.

In addition, more studies are needed to identify the best laboratory monitoring for renal dysfunction in patients on TDF. In a retrospective cohort study done on 356 HIV-infected patients in Thailand, TDF-associated renal dysfunction was assessed using 3 different methods: serum creatinine (SCr), calculated creatinine clearance (CCrCl), and eGFR. Significant change of SCr was detected in 5.3% of patients, in contrast to CCrCl (14.9%) and eGFR (17.7%). The investigators concluded that SCr is less sensitive than CCrCl and eGFR for detecting renal function decline in their cohort. This was likely because endogenous creatinine production is determined by muscle mass and dietary intake, which varies among different ethnic and age groups. In addition, a significant increase in SCr is detected only when GFR decreases to 60% of normal.[35]

The 24-hour urine collection is the standard method of quantifying proteinuria, although spot protein can be used to approximate the 24-hour proteinuria, as it is often difficult to obtain 24-hour protein collections in the outpatient setting. Spot protein is recommended for initial screening purposes with a 24 hour urine collection for confirmation. Furthermore, routine 24-hour urine collections are performed so infrequently clinical practice that few patients can be studied using this parameter in a retrospective study. Albumin/creatinine ratio and protein/creatinine ratio are alternative parameters that can be measured, but these are not routinely performed in our HIV clinic. Additionally, limitations associated with quantitative analyses include the variable fluctuations in urine protein, including hydration status, physical activity or illness, and menstrual blood, vaginal discharge or semen in the urine sample. [28] On the other hand, there is no significant difference in measurement of P/O-ratio from first void or random urine samples. [28] In our study, 80% of the subjects had abnormal P/O-ratios during the study period, and abnormal P/O-ratio was not associated with abnormal eGFR in this time frame, suggesting that abnormal P/O-ratio may be a very early biomarker of decreased renal function in HIV infected patients. However, we did identify significant associations with a number of clinically relevant risk factors for renal dysfunction on TDF.

Tubular dysfunction may exist in the absence of detectable glomerular dysfunction. TDF is eliminated in the urine by both glomerular filtration and proximal tubular secretion, so damage at either the glomerular or tubular level is significant. In a prospective observational study in Spain, 284 HIV patients were examined: 154 on TDF, 49 on other HAART regimens, and 81 drug-naïve. This study assessed both glomerular and tubular parameters of kidney function in HIV individuals. Glomerular parameters included plasma creatinine levels and creatinine clearance. Tubular parameters included glucosuria, hyperaminoaciduria, hyperphosphaturia, hyperuricosuria and beta-2-microglobulinuria. Although the 3 patient groups had no significant differences in glomerular parameters, up to 22% of HIV patients treated with TDF showed two or more abnormal tubular parameters. In contrast, this observation was found in only 6% of patients treated with other HAART regimens and in 12% of antiretroviral-naïve patients.[36]

Chronic tubulopathy in the absence of glomerular dysfunction is clinically relevant because a subset of patients may progress to renal insufficiency over time. In addition, long-term consequences of chronic hyperphosphaturia (a marker of tubular dysfunction) may lead to premature osteoporosis. Moreover, beta-2–microglobulin concentrations in urine are a sensitive marker of tubular damage. In the previously mentioned study, patients on TDF showed the greatest levels of beta-2–microglobulin in urine, which reinforces the potential for tubular toxicity of TDF. Some authors have proposed monitoring urine beta-2–microglobulin in patients on TDF to identify those at increased risk for renal injury.[36] Still others have demonstrated that low urine albumin-to-protein-ratio is a reliable marker of TDF nephrotoxicity. [37]

However, current DHHS guidelines have not included tubular parameter monitoring as part of their recommended guidelines for routine TDF monitoring. As a result, it is likely that these tests are underutilized by clinics that follow the DHHS guidelines for antiretroviral management. For example, our HIV clinic routinely performs screening urinalysis which includes a measure of proteinuria (P/O-ratio) but does not routinely request measures of tubular damage according to practice guidelines. Based on the results of this study, it is reasonable for HIV clinics to consider including more formal evaluation of tubular damage in routine monitoring urinalyses for patients on TDF.

Given ongoing concerns regarding potential nephrotoxicity of TDF and the need for continued nucleotide reverse transcription inhibitors in the backbone of cART regimens, tenofovir alafenamide fumarate (TAF), a new TFV prodrug, was recently FDA approved. TAF has been shown in clinical trials to be associated with fewer renal adverse effects compared to TDF. [38–41] However, TAF is currently only available in a co-formulation with elvitegravir, cobicistat and emtricitabine, which significantly limits the population who would be eligible to use this drug, either because of drug-drug interactions, co-morbidities or (more likely) cost. Therefore, TDF still remains an important component of antiretroviral regimens both in the United States and worldwide, underscoring the ongoing need for studies to determine optimal monitoring parameters to predict potential renal insufficiency.

### Limitations

The retrospective nature and small sample size of this study limit the ability to make accurate predictions regarding the associations of P/O-ratio and abnormal renal function. Many of the patients in our clinic come to us already on TDF-containing cART regimens; therefore identifying a control cohort not on a TDF-containing regimen was difficult, which was a limitation of the study. Additionally, baseline urinalyses to capture P/O-ratio prior to initiation of TDF were not available for every patient to determine a true incidence or time to event analysis of renal dysfunction as measured by P/O-ratios or eGFR. It would have been ideal to validate our P/O-ratio findings by comparing to protein-creatinine or 24hr proteinuria collection. However, this is difficult to achieve in a retrospective study if not part of routine practice. Nevertheless, future studies would certainly include a prospective analysis, identifying both glomerular and tubular measures of renal disease. Despite these limitations, the associations identified between duration of TDF therapy and P/O-ratio have important implications for patient care.

## Conclusions

Our study demonstrated that abnormal renal function, as measured by abnormal P/O-ratio, was common in HIV infected patients on TDF but was not associated with abnormal eGFR, suggesting that it may be a very early biomarker of decreased renal function in HIV infected patients. Duration of TDF use, age, diabetes and hypertension were associated with renal dysfunction in these patients; therefore, caution should be used when prescribing this medication in older patients or patients with hypertension or diabetes. Newer antiretrovirals provide an opportunity for alternative regimens with a tenofovir backbone; however these are likely to be out of reach to resource-poor communities. Therefore, because patients worldwide will likely still be on TDF-containing regimens, our study and future studies of TDF remain relevant to identify optimal parameters with which to monitor renal function in HIV patients, which could include both glomerular and tubular surrogates.
